# *atonal*- and *achaete-scute*-related genes in the annelid *Platynereis dumerilii*: insights into the evolution of neural basic-Helix-Loop-Helix genes

**DOI:** 10.1186/1471-2148-8-170

**Published:** 2008-06-09

**Authors:** Elena Simionato, Pierre Kerner, Nicolas Dray, Martine Le Gouar, Valérie Ledent, Detlev Arendt, Michel Vervoort

**Affiliations:** 1 Evolution et Développement des métazoaires, Centre de Génétique Moléculaire-UPR 2167 CNRS, 1, av. de la terrasse, 91198 Gif-sur-Yvette, France; 2 Belgian EMBnet Node – Laboratoire de Bioinformatique, Université Libre de Bruxelles, Institut de Biologie et de Médecine Moléculaires, Rue des Professeurs Jeener et Brachet 12, B-6041 Gosselies, Belgium; 3 Developmental Biology Unit, European Molecular Biology Laboratory, 69117 Heidelberg, Germany; 4 UFR Sciences du Vivant, Université Paris Diderot – Paris 7, 5, rue Marie-Andrée Lagroua Weill-Hallé, 75205 Paris Cedex 13, France

## Abstract

**Background:**

Functional studies in model organisms, such as vertebrates and *Drosophila*, have shown that basic Helix-loop-Helix (bHLH) proteins have important roles in different steps of neurogenesis, from the acquisition of neural fate to the differentiation into specific neural cell types. However, these studies highlighted many differences in the expression and function of orthologous bHLH proteins during neural development between vertebrates and *Drosophila*. To understand how the functions of neural bHLH genes have evolved among bilaterians, we have performed a detailed study of bHLH genes during nervous system development in the polychaete annelid, *Platynereis dumerilii*, an organism which is evolutionary distant from both *Drosophila *and vertebrates.

**Results:**

We have studied *Platynereis *orthologs of the most important vertebrate neural bHLH genes, i.e. *achaete-scute, neurogenin, atonal, olig*, and *NeuroD *genes, the latter two being genes absent of the *Drosophila *genome. We observed that all these genes have specific expression patterns during nervous system formation in *Platynereis*. Our data suggest that in *Platynereis*, like in vertebrates but unlike *Drosophila*, (i) *neurogenin *is the main proneural gene for the formation of the trunk central nervous system, (ii) *achaete-scute *and *olig *genes are involved in neural subtype specification in the central nervous system, in particular in the specification of the serotonergic phenotype. In addition, we found that the *Platynereis NeuroD *gene has a broad and early neuroectodermal expression, which is completely different from the neuronal expression of vertebrate *NeuroD *genes.

**Conclusion:**

Our analysis suggests that the *Platynereis *bHLH genes have both proneural and neuronal specification functions, in a way more akin to the vertebrate situation than to that of *Drosophila*. We conclude that these features are ancestral to bilaterians and have been conserved in the vertebrates and annelids lineages, but have diverged in the evolutionary lineage leading to *Drosophila*.

## Background

Neurogenesis is a complex process that involves the formation of a vast array of neuronal and glial cell types that must be produced in the correct numbers and at appropriate positions. Genetic and molecular studies mainly conducted in *Drosophila *and vertebrates have shown that genes encoding transcription factors of the basic Helix-Loop-Helix (bHLH) class play pivotal roles in various steps of neurogenesis, including commitment of neural precursors (proneural function), specification of particular neuronal identities, and neuronal differentiation [1-5]. Most of the genes encoding bHLH transcription factors and which are involved in neural development (hereafter named neural bHLH genes), belong to five of the numerous phylogenetically-defined bHLH families, *achaete-scute *and four families of *atonal*-related genes, *neurogenin*, *atonal*, *olig (oligo)*, and *NeuroD *[2,6]. While some of the neural bHLH genes show strikingly similar functions in *Drosophila *and vertebrates [2,3,7], there are also profound differences between them [1,4].

First, in vertebrates, genes of the *neurogenin *family (*ngn1*, *ngn2*, and *ngn3*) are required for the formation of the precursors of many neural cells of both the Peripheral and Central Nervous Systems (PNS and CNS) [8-10] while their single *Drosophila *ortholog, *tap/biparous*, has no proneural role and is expressed in a few differentiating neural cells [11,12]. In *Drosophila*, the main proneural bHLH genes for the CNS belong to the *achaete-scute *family and are also involved, together with *atonal *family genes, in the formation of the sensory organs [2,3,13,14]. Vertebrate *achaete-scute *and *atonal *genes probably also have proneural functions but in a much more limited set of cells, in particular in the CNS [1,2,4].

Second, vertebrate proneural genes contribute to the specification of progenitor-cell identity [2,4,5]. A clear example of such a function is provided by the dorsal embryonic spinal cord, in which *Math1 *(*atonal *family), *ngn1*, and *Mash1 *(*ascl1*; *achaete-scute *family) are required for the correct specification of discrete dorsoventral progenitor domains that produce distinct types of interneurons [15-18]. *Mash1 *has also been shown to have instructive roles in the specification of noradrenergic, GABAergic, and serotonergic neurons in various positions in the brain and the spinal cord [19-22]. Finally, *ngn2 *has a key role for motor neurons formation in the ventral spinal cord [23,24]. Such important roles in neuronal specification for proneural bHLH genes in the CNS are not found in *Drosophila *[1,4].

Third, bHLH genes that have important functions during vertebrate neurogenesis do not have orthologs in *Drosophila*. Many vertebrate neurons require the function of genes, which belong to the *NeuroD *family, for their proper differentiation and survival [25-27]. Genes of the *olig *family (*olig1*, *olig2*, and *olig3*) have key roles in the specification of motor neurons, dorsal interneurons, and oligodendrocytes in the vertebrate CNS [18,23,24,28-30]. *olig *and *NeuroD *genes do not exist in *Drosophila *(the gene known in *Drosophila *as *Dm-oli *is in fact an ortholog of the vertebrate *Beta3 *genes) [6,31].

Given these differences and in order to decipher which aspects of the functions of neural bHLH genes are ancestral to bilaterians and which are derived characters specific to some bilaterian lineages, we have isolated and studied these genes in the polychaete annelid, *Platynereis dumerilii*, which belongs to a different branch (Trochozoa) of the bilaterians tree than *Drosophila *(Ecdysozoa) and vertebrates (Deuterostoma) and is therefore evolutionary distant to both these organisms [32]. In addition, *Platynereis *is considered to have retained some bilaterian ancestral features, making it a useful model for comparative developmental biology [e.g. [33-35]]. Here, we report the expression patterns of *Platynereis *orthologs of the most important neural bHLH genes, including the *olig *and *NeuroD *genes not found in *Drosophila*. Our data suggest that *Platynereis *bHLH genes have both proneural and neuronal specification functions, in a way more akin to the vertebrate situation than to that of *Drosophila*. These data indicate that these functions were already established in *Urbilateria*, the last common ancestor of all bilaterians.

## Results

### Brief overview of the formation of the Platynereis larval nervous system

*Platynereis *displays an indirect development which gives rise to a ciliated trochophore larva that subsequently metamorphoses into a juvenile worm [36]. The formation of the *Platynereis *larval trunk nervous system has been thoroughly described, up to the late trochophore stage (metatrochophore; 48 to 55 hours post fertilization, hpf), using whole-mount *in situ *hybridization (WMISH) with RNA antisense probes corresponding to *Platynereis *neuronal differentiation genes, such as *elav *(*Pdu-elav*), *synaptotagmin *(*Pdu-syt)*, *Tryptophane Hydroxylase *(*Pdu-TrpH*), and *Vesicular Acetylcholine Transporter *(*Pdu-VAchT*) [35]. In order to give an overview of larval neurogenesis, which is important to understand the next parts of this article, we show here some WMISH for these previously characterized genes in 24 hpf to 55 hpf larvae and extend the published description by looking at juvenile worms (72 hpf).

**Figure 1:**
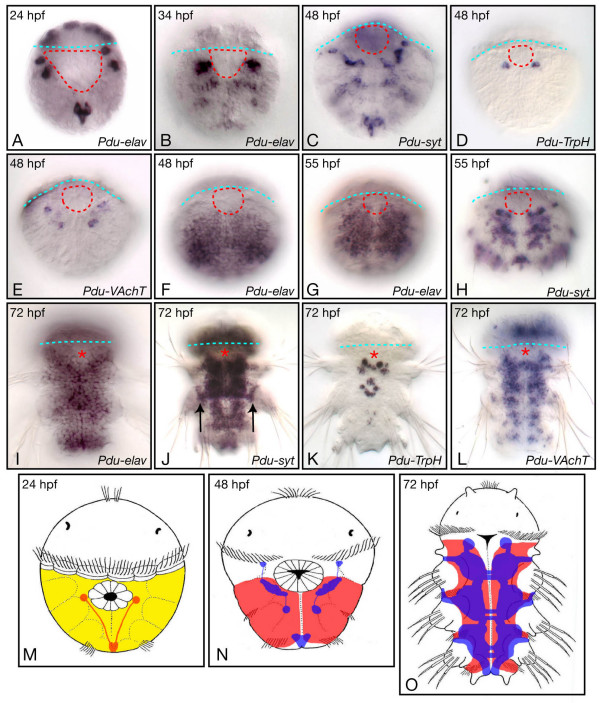
**Overview of formation the trunk nervous system in *Platynereis***. Expression of *Pdu-elav*, *Pdu-syt*, *Pdu-TrpH*, and *Pdu-VAchT*, as determined by WMISH, on a selection of larval stages is shown. All pictures are ImageJ projections and are ventral views (anterior up). The blue dotted lines indicate the position of the prototroch (a ring of ciliated cells involved in the locomotion of the larva) and therefore the separation between the prospective head (up) and trunk (down) regions. The position of the mouth region is indicated either by dotted red lines or a red asterisk, depending on the stage. The formation of the larval neurons is shown in A to E. A few neurons with stereotyped positions differentiate between 24 h to 48 h as seen by the expression of *Pdu-elav *and *Pdu-syt *(A-C). A single pair of neurons, located close to the mouth region, is serotonergic as seen by the expression of *Pdu-TrpH *(D), a few other neurons are cholinergic (as seen by the expression of *Pdu-VAchT*; E), and the other ones are of unknown identity. F to L depict the formation of the juvenile worm nervous system. See the main text for details. Arrows in J point to peripheral neurons associated with the parapodes. M to O are schematic drawings of the organization of the nervous system of 24 hpf, 48 hpf, and 72 hpf larvae. The neuroectodermal cells are indicated in yellow, the cells expressing *Pdu-elav *in red, and the *Pdu-syt*-expressing neurons in blue.

A simple larval nervous system first differentiates during the early (24 hpf) to late trochophore (48 hpf) stages: a few cells expressing *Pdu-elav *are observed on the ventral side of the 24 hpf and 34 hpf larvae (Figure 1A,B) and these cells give rise to two bilateral anterior groups and one posterior group of *Pdu-syt*-expressing neurons (Figure 1C). Different neuronal subtypes can be identified, such as serotonergic (identified as cells expressing *Pdu-TrpH*) and cholinergic (identified as cells expressing *Pdu-VAchT*; Figure 1D,E). From 48 hpf, a large number of cells enter neural differentiation and express first *Pdu-elav *(Figure 1F,G) and then *Pdu-syt *(Figure 1H). These neurons form the ventral nerve cord (VNC) of the 72 hpf juvenile worm (Figure 1I,J). Many of the formed neurons are cholinergic neurons as seen by the massive expression of *Pdu-VAchT *(Figure 1L) in the VNC, except in its medialmost part where several serotonergic neurons differentiate (expression of *Pdu-TrpH*; Figure 1K). Outside the VNC, peripheral neurons are also found mainly associated with the appendages (the parapodes; arrows in Figure 1J).

### Identification of Platynereis atonal- and achaete-scute-related bHLH genes

**Figure 2:**
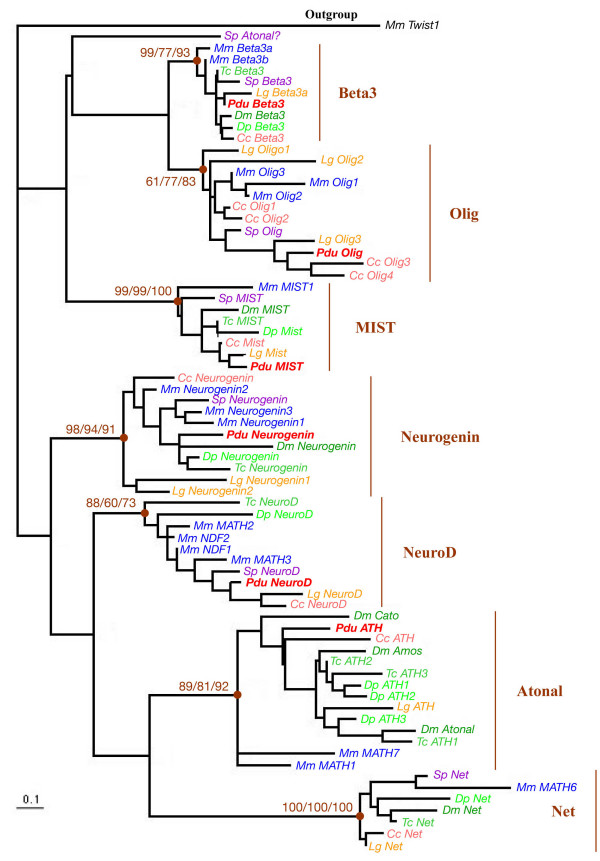
**Phylogenetic analysis of the *Platynereis atonal*-related bHLH genes**. The phylogenetic tree has been constructed by Maximum Likelihood (ML) as described in the Methods section. Similar tree topologies were obtained using other phylogenetic reconstruction methods (not shown). The different groups of orthology are indicated (for more details, see [31]). Statistical supports for the internal branches that define these groups are indicated (first number: bootstrap support in Neighbour-joining (NJ) analysis; second number: bootstrap support in ML analysis; third number: posterior probability in Bayesian inference analysis). The tree has been rooted using a non-*atonal*-related (*twist*) bHLH gene as outgroup. *Platynereis *genes are indicated in bold red. Species abbreviations: *Cc*: *Capitella spI *(annelid); *Dm*: *Drosophila melanogaster *(insect); *Dp*: *Daphnia pulex *(crustacean); *Lg*: *Lottia gigantea *(mollusk); *Mm*: *Mus musculus *(vertebrate); *Pdu*: *Platynereis dumerilii *(annelid); *Sp*: *Strongylocentrotus purpuratus *(echinoderm); *Tc*: *Tribolium castaneum *(insect).

**Figure 3:**
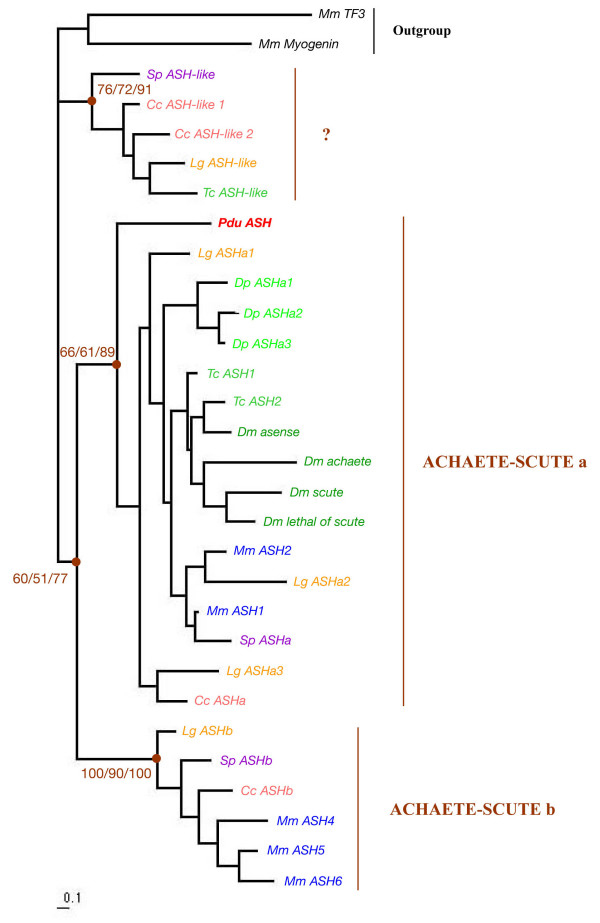
**Phylogenetic analysis of the *Platynereis achaete-scute*-related bHLH gene**. The phylogenetic tree has been constructed by Maximum Likelihood (ML) as described in the Methods section. Similar tree topologies are obtained using other phylogenetic reconstruction methods (not shown). Statistical supports for the internal branches that define these groups are indicated (first number: bootstrap support in Neighbour-joining (NJ) analysis; second number: bootstrap support in ML analysis; third number: posterior probability in Bayesian inference analysis). The tree has been rooted using non-*achaete-scute *bHLH genes as outgroup. The *Platynereis *gene is indicated in bold red. The "?" indicates a group of divergent *achaete-scute*-like genes found in some species and that cannot be related to either of the two bilaterian *achaete-scute *families (see [31] for more details). Species abbreviations are as in Figure 2.

By sequence similarity searches on an expressed sequence tag (EST) collection (40,000 ESTs from normalized cDNA libraries of mixed larval stages, corresponding to more than 10,000 unigene clusters) [34], we identified several *Platynereis dumerilii *bHLH genes among which some show strong sequence similarity to either *achaete-scute*- or *atonal*-related genes. The predicted amino acid sequence of the bHLH domains were aligned with those of a sample of metazoan bHLH genes identified in a previous study [31]. Multiple phylogenetic reconstructions show that we identified orthologs of the *neurogenin/biparous, achaete-scute/ASCL*/*ASH*, *olig*, and *NeuroD *genes (Figures 2 and 3). We named these genes *Pdu-Ngn, Pdu-ASH*, *Pdu-Olig*, and *Pdu-NeuroD*, respectively. For each family, we found a single *Platynereis *member but we cannot exclude that additional members do exist, although duplicated evolutionary-conserved genes are rare in *Platynereis *[34]. We also included in our analysis the previously identified *atonal *ortholog (*Pdu-ATH*; not found in the EST collection) [35,37] in order to study *Platynereis *representatives for all the main families of bHLH genes involved in neural determination and specification in vertebrates and *Drosophila *[2,4]. We also identified *Beta3 *and *mist *genes in *Platynereis *(Figure 2), but these genes were not further characterized as their *Drosophila *and vertebrate orthologs do not have well defined functions in neurogenesis. We used WMISH to monitor the expression of *Pdu-Ngn, Pdu-ASH*, *Pdu-Olig, Pdu-ATH*, and *Pdu-NeuroD *during *Platynereis *development and focused on possible expressions during trunk nervous system formation for which a good characterization exists [35]. All the genes are also expressed in the head, probably in the brain and/or sensory organs, but these expressions were not further characterized.

### Platynereis atonal- and achaete-scute-related bHLH genes are expressed during trunk nervous system formation

**Figure 4:**
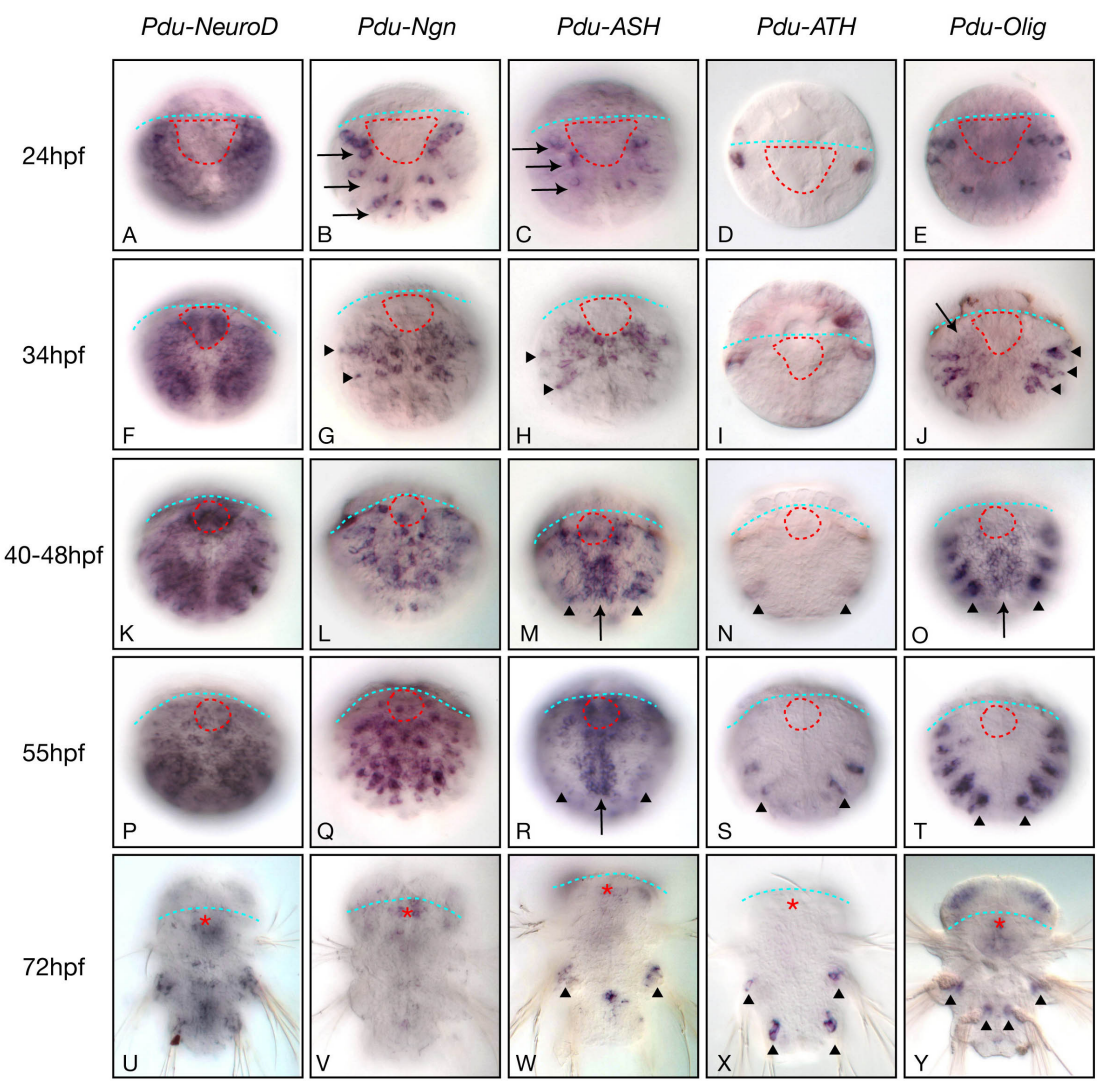
**Expression of *Platynereis *neural bHLH genes during trunk neurogenesis**. Expression of *Pdu-NeuroD*, *Pdu-neurogenin (Pdu-Ngn)*, *Pdu-achete-scute (Pdu-ASH)*, *Pdu-Olig*, and *Pdu-atonal *(*Pdu-ATH*), as determined by WMISH, on a selection of larval stages is shown. Most of the images are ImageJ projections and all images are ventral views (anterior up). Labels are as in Figure 1. See the main text for the detailed descriptions of the expression patterns. In (B) and (C), arrows point to the groups of cells expressing *Pdu-Ngn *or *Pdu-ASH *and whose positions correspond to the neurons that will latter differentiate (compare with Figure 1B,C). In (G) and (H), the arrowheads point to putative PNS precursor cells. In (J), the arrow points to a weak medial expression of *Pdu-Olig *and arrowheads to its more lateral expression domains that form three bilateral small stripes. In (M, N, O, R, S, T), the arrow points to the medial expression and the arrowheads to the more lateral expression domains that lie outside the VNC. In (W-Y), arrowheads point to expressions in cells associated with the parapodes. Labelling associated with the parapodes in (U) is probably background (as seen at higher magnification).

**Figure 5:**
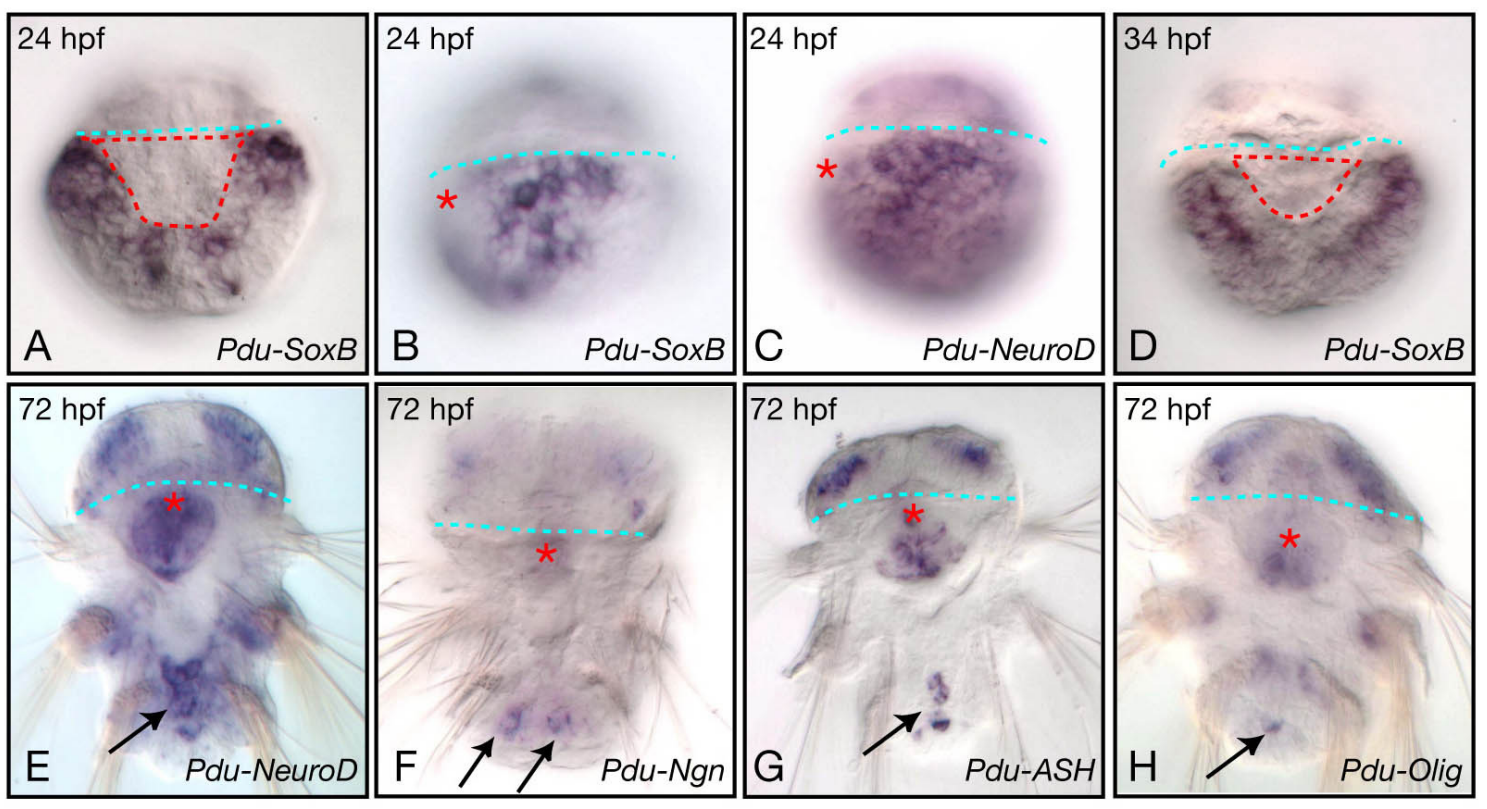
**Additional expressions of *Platynereis *neural genes during trunk neurogenesis**. A, D, E-H are ventral views; B, C are lateral views (ventral on the left). Most of the images are ImageJ projections. Labels are as in Figure 1. E-H correspond to more internal views than those of Figure 4U-Y which are superficial views. See the main text for details. Arrows in E-H point to internal cells expressing neural bHLH genes and that may belong to the posterior growth zone [41,42].

The five bHLH genes are already expressed in 24 hpf larvae. *Pdu-NeuroD *is expressed in a broad ventral ectodermal domain (Figure 4A) which corresponds to the larval neuroectoderm. This expression domain is similar to that of *Platynereis *orthologs of bilaterian conserved neuroectodermal genes, such as *SoxB *(Figure 5A–C and P.K. et al., unpublished). *Pdu-Ngn *and *Pdu-ASH *are expressed in a few cells located on the ventral side of the larvae, in particular around the stomodaeal area (Figure 4B,C). This expression nicely prefigures the distribution of differentiating neurons that is observed in slightly later stages (Figure 1B,C), suggesting that the two genes are expressed in precursors of the larval central nervous system. *Pdu-ATH *and *Pdu-Olig *are expressed in a few lateral cells we were unable to identify (Figure 4D,E).

In 34 hpf larvae, *Pdu-NeuroD *is still expressed in a broad ventral ectodermal domain (Figure 4F) which includes the prospective VNC region in which *Pdu-Ngn *and *Pdu-ASH *become widely expressed (Figure 4G,H). At this stage, the expression of *Pdu-SoxB *strongly decreases in the same region (Figure 5D). In vertebrates, the transition from neuroectodermal cells to committed progenitors is linked to the activation of the expression of bHLH proneural genes and a concomitant repression of the expression of *SoxB *genes [e.g. [2,38]]. Our data suggest that a similar transition occurs around 34 hpf in the *Platynereis *larvae. However, at this stage, only very few cells express *Pdu-elav *(Figure 1B), indicating that the *Pdu-Ngn *and *Pdu-ASH *expressing precursors are still not engaged towards differentiation. Both *Pdu-Ngn *and *Pdu-ASH *are also expressed in more lateral cells that may correspond to peripheral nervous system precursors (Figure 4G,H). *Pdu-ATH *and *Pdu-Olig *continue to be expressed in lateral cells, with *Pdu-Olig *expressed in three bilateral small stripes (Figure 4I,J).

**Figure 6:**
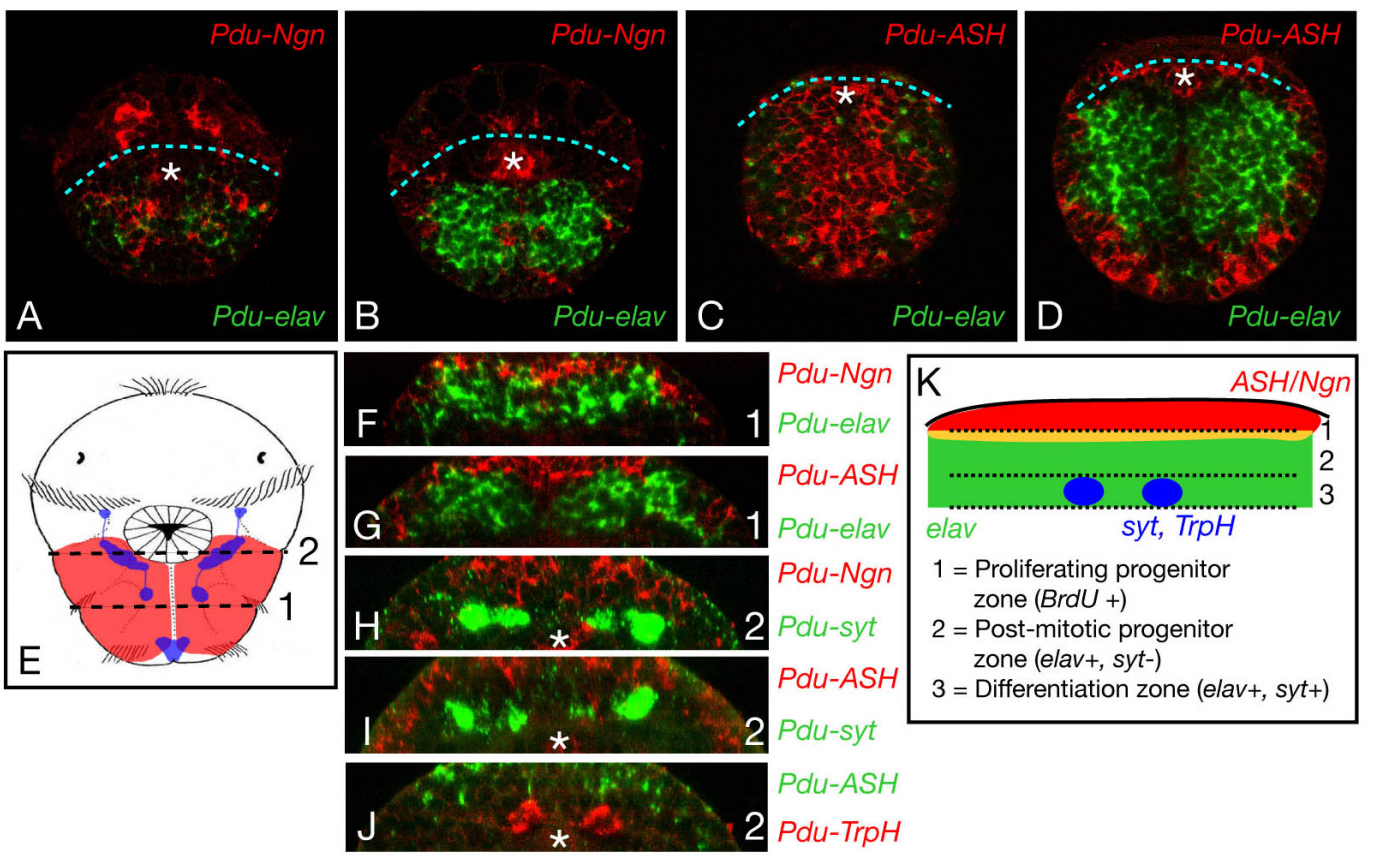
**Position of the *Pdu-Ngn*- and *Pdu-ASH*-expressing cells with respect to the apicobasal organization of the prospective ventral nerve cord region**. Confocal picures of double WMISH are shown. The probes which have been used are indicated on the pictures. A and C are superficial plans, B and D more internal ones. *Pdu-Ngn *and *Pdu-ASH *are mainly expressed in superficial cells while *Pdu-elav *is mainly expressed in more profound cells. F to J are virtual cross-sections, sections have been made at two anteroposterior levels (1 and 2) as indicated on a schematic drawing (E). Apical is up, basal is down. *Pdu-Ngn *and *Pdu-ASH *are mainly expressed in the apicalmost layer of cells of the prospective VNC while *Pdu-elav*, *Pdu-syt*, and *Pdu-TrpH *are expressed in more basal cells. Some internal cells also express *Pdu-Ngn *(H) and *Pdu-ASH *(not shown) and on some confocal sections we can see rare cells co-expressing *Pdu-Ngn *or *Pdu-ASH *and *Pdu-elav*. Such co-expressions are never observed with *Pdu-syt *or *Pdu-TrpH*. The white asterisk indicates an expression in the stomodeum. K summarizes the apicobasal layering of the *Platynereis *prospective VNC based on the data published by Denes et al. [35] and includes the expression of *Pdu-Ngn *and *Pdu-ASH *defined in this study. Apical is up, basal is down. *Pdu-elav *expression domain is in green, *Pdu-ASH *and *Pdu-Ngn *expression domain is in red, and the yellow region indicates the zone of overlap of these expression domains.

In 40 hpf to 55 hpf larvae, *Pdu-NeuroD *continues to be broadly expressed in the ventral ectoderm (Figure 4K,P). Expression of *Pdu-NeuroD *is restricted to the superficial layer of the ectoderm (not shown). From 40 hpf to 55 hpf larvae, *Pdu-Ngn *is largely expressed in the whole prospective VNC region as well as in some more lateral cells (Figure 4L,Q). *Pdu-Ngn *is expressed in a salt and pepper manner, with highly-expressing cells interspersed with weakly-expressing ones. In 40 hpf and 48 hpf larvae, Denes et al. [35] showed that the prospective *Platynereis *VNC region is multilayered with, from superficial (apical) to more internal (basal), a proliferating progenitor zone (a single layer of BrdU incorporating cells), a post-mitotic progenitor zone (*Pdu*-*elav *positive, *Pdu-syt *negative cells), and a differentiation zone (*Pdu*-*elav *positive, *Pdu-syt *positive cells). To define in which zone(s) of the prospective VNC *Pdu-Ngn *is expressed, we performed double WMISH [39] for *Pdu-Ngn*, on one hand, and *Pdu-elav *or *Pdu-syt*, on the other hand, and visualized the labellings with confocal microscopy (Figure 6). We found that *Pdu-Ngn *is mainly expressed in superficial cells in contrast to *Pdu-elav *and *Pdu-syt *(Figure 6A,B). We used 3D reconstructions of confocal stacks to perform virtual cross-sections of the VNC region (Figure 6F,H): we found that *Pdu-Ngn *is expressed in the apicalmost cells of the prospective VNC with very little overlap with *Pdu-elav *and no overlap with *Pdu-syt*. We therefore conclude that *Pdu-Ngn *is expressed in undifferentiated neural precursors and mainly in the proliferating ones.

*Pdu-ASH *and *Pdu-Olig*, at 40 and 48 hpf, are expressed in the medialmost part of the prospective VNC region as well as in more lateral cells (Figure 4M,O). As for *Pdu-Ngn*, *Pdu-ASH *and *Pdu-Olig *are expressed in proliferating progenitors, as determined by double WMISH (Figure 6C,D,G,I and not shown). The expression of *Pdu-ASH *and *Pdu-Olig *in the medialmost part of the prospective VNC region is similar to that of the *Platynereis NK2.2 *gene and corresponds to the region from which serotonergic neurons will emerge (Figure 1K) [35]. We confirmed by double WMISH that the serotonergic neurons (*Pdu-TrpH*-expressing cells) are located below the *Pdu-ASH*-expressing precursors (Figure 6J). At 55 hpf, *Pdu-ASH *shows the same expression pattern than in the previous stages, but *Pdu-Olig *is no more expressed in the prospective VNC region (Figure 4R,T). In 40 hpf to 55 hpf larvae, *Pdu-ATH *is only expressed in a few cells located on both sides of the prospective VNC region (Figure 4N,S).

At 72 hpf, the five genes are expressed in more limited sets of cells in the VNC region and in cells associated with the parapodes (Figure 4U–Y). In addition, *Pdu-Ngn*, *Pdu-ASH, Pdu-Olig*, and *Pdu-NeuroD *are expressed in posteriorly-located internal cells (Figure 5E–H) whose position roughly corresponds to that of cells expressing *Platynereis hunchback *[40] and stem-cells markers, such as *Platynereis piwi *and *vasa *[41]. The cells expressing *Pdu-Ngn*, *Pdu-ASH, Pdu-Olig*, and *Pdu-NeuroD *may therefore belong to the posterior subterminal growth zone that will allow the posterior addition of new segments to the existing ones (and the corresponding elongation of the VNC) in a sequential manner throughout most of the life of the animal [41,42].

## Discussion

### Olig and NeuroD genes belong to the ancestral bilaterian toolkit of neural developmental genes

In this article, we report, for the first time, the characterization of several *atonal*- and *achaete-scute*-related bHLH genes from a lophotrochozoan species, the annelid *Platynereis dumerilii*. Our phylogenetic analyses demonstrate that we have identified *neurogenin*, *achaete-scute*, *olig*, and *NeuroD *orthologs in addition to a previously characterized *atonal *gene (Figures 2 and 3). *neurogenin*, *achaete-scute*, and *atonal *genes have been found in many diverse species, including *Drosophila melanogaster*, *Caenorhabditis elegans*, and several vertebrates, and shown to be involved in neural development in all these species [e.g. [2,7,31]]. We found a similar situation in *Platynereis *as the three genes display specific expressions during neurogenesis (see below for further discussion), confirming their evolutionary-wide implication in neurogenesis in bilaterian animals.

*NeuroD *genes have been shown to be important neuronal differentiation genes in vertebrates (see introduction). A putative *NeuroD *gene (named *cnd-1 *but whose orthology relationship with the vertebrate *NeuroD *genes is only poorly supported; [43]) has been described in the nematode *Caenorhabditis elegans *and is involved in several steps of the formation of the motor neurons [44]. *NeuroD *genes cannot be found in the genomes of *Drosophila melanogaster *and of the urochordate *Ciona intestinalis *[6,45]. Our phylogenomic analysis (Figure 2) indicates that *NeuroD *genes are in fact widely found in bilaterians and that their absence in *Drosophila melanogaster *and *Ciona intestinalis *results from a rather specific loss in these species. We found the *Platynereis NeuroD *gene to have a broad and early neuroectodermal expression, which is completely different from the neuronal expression of vertebrate *NeuroD *genes and the neural subtype-specific expression of *Caenorhabditis cnd-1*. It is therefore difficult to infer from these data any putative ancestral expression or function of *NeuroD*. More precise inference will await additional data from other species.

*Olig *genes were thought to be vertebrate-specific genes as they are not found in the *Drosophila melanogaster*, *Caenorhabditis elegans*, and *Ciona intestinalis *genomes [6,45]. In fact, *olig *genes are found in several non-vertebrate species (Figure 2). However, we cannot find such genes in any of the sequenced arthropod and nematode genomes [[31], E.S. and M.V., unpublished observations], suggesting that *olig *genes have been lost quite early during the evolution of these phyla. We found the *Platynereis olig *gene to be specifically expressed during nervous system formation and that its expression shows similarities to that of vertebrate *olig *genes (see below). We therefore conclude that *olig*, together with *neurogenin*, *achaete-scute*, *atonal*, and *NeuroD *genes belong to the ancestral bilaterian toolkit of neural developmental genes.

### Vertebrate-like expression of Platynereis neurogenin suggests a major proneural role and provides insights into the evolution of the proneural function in bilaterians

One striking difference between vertebrate and *Drosophila *neurogenesis is the differential use of *neurogenin *genes in these species. Indeed, *neurogenin *genes are the key proneural genes in the vertebrate CNS while it is not the case for their *Drosophila *counterpart (see introduction). In *Drosophila*, and probably more generally in arthropods, the proneural function in the CNS is mainly performed by the *achaete-scute *genes (see introduction and [46]). These genes have a much more limited proneural functions in vertebrates (see introduction). Given these differences, it is challenging to infer what was the ancestral situation, i.e. what were the main proneural genes acting in *Urbilateria*, the last common ancestor of all bilaterians. Our data on *Platynereis *help to answer this question.

We found that *Platynereis neurogenin *(*Pdu-Ngn*) has an expression suggestive of a wide proneural function in the developing trunk CNS which is similar to that of the vertebrate *neurogenin *genes. Indeed, *Pdu-Ngn *expression arises at early stages of neural development, overlapping that of the neuroectodermal marker *SoxB*, and preceding the expression of the differentiation marker *elav*. The expression domain of *Pdu-Ngn *is large and covers the whole prospective CNS region (ventral part of the trunk ectoderm) as well as more lateral regions which probably correspond to the PNS. *Pdu-Ngn *is expressed in a salt and pepper manner which is often found for proneural genes as a consequence of lateral inhibition processes (reviewed in [1,2]). Finally, *Pdu-Ngn *is expressed in the apicalmost part of the forming CNS, which has been shown to include proliferative neuroectodermal cells and neural precursors [35]. *Pdu-Ngn *is therefore expressed at the right time, the right place, and the right manner to be the major proneural gene for the formation of the *Platynereis *trunk nervous system, like its orthologs in vertebrates. This similarity between vertebrates (deuterostomes) and an annelid (a protostome) suggests that the broad proneural function of *neurogenin *genes is ancestral to bilaterians and has been lost in the evolutionary lineage leading to present-day insects (or even arthropods). This does not necessarily mean that proneural function of *achaete-scute *genes is a derived character: indeed, such function is found in vertebrates and in arthropods, and the expression of *Pdu-ASH *is not incompatible with this function, as it is expressed in early stages of VNC formation (although only in a subset of the *Pdu-Ngn*-expressing cells – an expression that is, to our opinion, more consistent with a role in neural specification). It is therefore conceivable that both *neurogenin *and *achaete-scute *were acting as proneural genes in *Urbilateria*, the former being the predominant one.

### Platynereis bHLH genes may have neural specification functions in the CNS – evolution of neural specification in bilaterians

Another striking difference between vertebrates and *Drosophila *is the differential use of bHLH genes in neural subtype specification in the CNS. In vertebrate, neural bHLH genes such as *olig *and *achaete-scute *genes are important to specify many types of neural cells which are produced in the CNS, while it is not the case in the *Drosophila *CNS (although a single study has suggested that *achaete-scute *genes may be involved in the specification of a limited set of cells in the CNS [47]; but see [1]). This raises the possibility that the functions of bHLH genes in neural subtype specification may largely represent vertebrate innovations. Alternatively, they could be bilaterian ancestral functions that have been lost in the evolutionary lineage leading to *Drosophila*. We found that two *Platynereis *bHLH genes, *Pdu-ASH *and *Pdu-Olig*, are expressed in a way suggestive of an involvement in neural specification in the CNS. Both genes are expressed in the medialmost part of the CNS, they are first co-expressed and in a second time, only *Pdu-ASH *continues to be expressed. Interestingly, these expression profiles coincide with those of vertebrates *olig *and *achaete-scute *genes which are also expressed in medial parts of the neural tube (reviewed in [5]). Importantly, this similarity is probably meaningful as the overall organization of the *Platynereis *CNS bears striking resemblances with that of vertebrates, as seen by the conserved expression domains of many neural patterning and differentiation genes between *Platynereis *and vertebrates [35]. The cells expressing *Pdu-ASH *and *Pdu-Olig *correspond to a domain of the VNC characterized by the co-expression of *Platynereis NK2.2 *and *NK6 *genes [35]. In vertebrates, such as mammals, *Ascl1/Mash1 *is also expressed in a *NK2.2/NK6 *positive medial domain (p3 domain in the spinal cord and its topological equivalent in the hindbrain, PMNv) and controls the formation of serotonergic neurons from this domain in the hindbrain [21,22]. Strikingly, serotonergic neurons in *Platynereis *also emerge from a medial domain of the CNS, corresponding to cells expressing *NK2.2 *and *achaete-scute*. We therefore suggest that *Pdu-ASH *is involved in the specification of serotonergic neurons in *Platynereis *and that this may represent an ancestral function of *achaete-scute *genes in bilaterians.

In vertebrates, such as mammals, *olig *genes have important roles in neural specification. *olig2 *gene is expressed in the so-called pMN domain of the spinal cord, located slightly more laterally than the p3 domain (see Introduction and [5]). This domain corresponds to a *NK6*/*Pax6*-positive domain from which originate *Hb9*-positive cholinergic motor neurons and oligodendrocytes, the specification of both of these cells types being controled by *olig2*. This *NK6*/*Pax6*-positive domain also exists in *Platynereis *and, as in vertebrates, *Hb9*-positive cholinergic neurons emerge from this domain [35]. *Pdu-Olig *is, however, not expressed in this domain (it is expressed in the more medial *NK6*/*NK2.2*-positive domain) and is therefore much probably not involved in motor neurons specification. However its co-expression with *Pdu-ASH *at some but not all stages of VNC formation, in the *NK6*/*NK2.2*-positive domain, suggests it may contribute to the diversification of neural cell types from this domain. We suggest that different neural cell types may form in the prospective *Platynereis *VNC first from the *Pdu-ASH*/*Pdu-Olig*-positive cells and then from the *Pdu-ASH*-positive (*Pdu-Olig*-negative) cells. This suggestion is based on the fact that, in vertebrates, different combinations of neural bHLH have been shown to control the formation of different cell types from a single medio-lateral domain [5,48].

Our data suggest that some neural bHLH genes are involved in neural subtype specification in *Platynereis*, like in vertebrates, and that this may therefore represent an ancestral feature of bilaterians. In insects, such as *Drosophila*, the specification functions of neural bHLH genes (and even some genes such as *Olig*) have been lost. This may be related to the fact that insects have evolved a divergent way to pattern their CNS: while in vertebrates and *Platynereis *the CNS is subdivided into large domains from which emerge specific neural cell types, insects have shifted to a mainly cell-to-cell-based process in which neuroblasts (even neighbouring ones) will express different combinations of developmental genes that control their identity.

## Conclusion

We have identified, for the first time in a lophotrochozoan species, *Platynereis dumerilii*, orthologs of the most important neural bHLH genes known in vertebrates, including the *Olig *and *NeuroD *genes not found in *Drosophila*. We have performed a detailed analysis of the expression patterns of these *Platynereis *bHLH genes and we show that all these genes are expressed during neurogenesis. Our analysis suggests that the *Platynereis *bHLH genes have both proneural and neuronal specification functions, in a way more akin to the vertebrate situation than to that of *Drosophila*. Our data suggest that these functions are ancestral to bilaterians.

## Methods

### Breeding culture, embryo collection, whole mount in situ hybridization (WMISH), microscopy, and image processing

Animals were obtained from a breeding culture established in Gif-sur-Yvette according to the protocol of Fisher and Dorresteijn [49]. Larvae collection and fixation, as well as WMISH, were done as previously described [39,50]. In the case of the double WMISH, one of the probes was revealed using tyramide signal amplification (fluorescent dye) and the other using the classical NBT/BCIP reaction. The NBT/BCIP staining was visualized by reflection confocal laser scanning microscopy [39]. Labeled embryos picture Z-stacks were manually taken on a Leica bright-field microscope and Z-projection images were made using ImageJ 1.36b. Confocal pictures were taken on a Leica Sp2 confocal microscope and images were 3D reconstructed with Metamorph.

### Isolation of Platynereis bHLH genes and phylogenetic analyses

*Platynereis *bHLH genes were identified by BLAST searches against an EST collection [34]. The coding sequences of the different genes were amplified using SMART™ RACE cDNA amplification procedures with gene-specific primers (whose sequences are available upon request). PCR-products were TA cloned into the PCR2.1 vector (Invitrogen), sequenced on an ABI automated sequencer, and used as template to produce labeled antisense RNA probes for WMISHs. Accession numbers for the newly cloned genes are FM163169 to FM163172.

Multiple sequence alignments were built using ClustalW [51] using a large set of bHLH domains derived from Simionato et al. [31] in addition to those encoded by the isolated *Platynereis *genes. Neighbour-joining (NJ) reconstructions were performed with the PAUP 4.0 program using the BioNJ algorithm and 10,000 bootstrap replicates [52,53]. Maximum likelihood (ML) analyses were performed with PHYML using the Jones, Taylor, Thornton (JTT) model of amino acid substitutions [54] and 150 bootstraps to assess the statistical reliability of the obtained internal branches. Bayesian inference was performed using the Markov chain Monte Carlo method as implemented in the MRBAYES (version 3) package [55]. We used the JTT substitution frequency matrix with among-sites rate variation modelled by means of a discrete γ distribution with four equally probable categories. Two independent Markov chains were run, each containing 2,000,000 Monte Carlo steps. One out of every 250 trees was saved. The trees obtained in the two runs were meshed and the first 25% of the trees were discarded as 'burnin'. Marginal probabilities at each internal branch were taken as a measure of statistical support. All the alignments and the trees are available upon request.

## Authors' contributions

ES participated in the cloning of the genes and carried out most *in situ *hybridizations; PK participated in the cloning of the genes and carried out some *in situ *hybridizations; ND carried out the double *in situ *hybridizations and confocal imaging; MLG participated in the cloning of the genes and *in situ *hybridizations; VL participated in BLAST searches and phylogenetic analyses; DA participated in the design of the study and provided essential sequence data and materials; MV conceived the study, participated in BLAST searches, performed the phylogenetic analyses, and drafted the manuscript. All authors have read and approved the final manuscript.
